# Adjuvant chemotherapy in stage II and III colon cancer: the role of the “budding and TILs-(tumor-infiltrating lymphocytes) combination” as tumor-host antagonists

**DOI:** 10.1007/s00384-021-03896-9

**Published:** 2021-03-20

**Authors:** Corinna Lang-Schwarz, Balint Melcher, Theresa Dregelies, Zahra Norouzzadeh, Stefanie Rund-Küffner, Klaus Lang-Schwarz, Michael Vieth, William Sterlacci

**Affiliations:** 1 Institute of Pathology, Klinikum Bayreuth GmbH, Preuschwitzer Str. 101, 95445 Bayreuth, Germany; 2 Institute of Pathology, Koblenz, Franz-Weis-Str. 13, 56073 Koblenz, Germany; 3 Department of Internal Medicine, Sana Klinik Pegnitz, GmbH, Langer Berg 12, 91257 Pegnitz, Germany; 4 Department of Anesthesiology, Klinikum Bayreuth GmbH, Preuschwitzer Str. 101, 95445 Bayreuth, Germany; 5 Institute of Pathology, Friedrich-Alexander-University Erlangen-Nuremberg, Krankenhausstr. 8-10, 91054 Erlangen, Germany

**Keywords:** Budding, Tumor-infiltrating lymphocytes (TILs), Colon cancer, Chemotherapy

## Abstract

**Purpose:**

To analyze the influence of adjuvant chemotherapy on the combination of tumor budding and tumor-infiltrating lymphocytes (TILs) in stage II and III colon cancer and to elucidate its potential value for adjuvant treatment decisions.

**Methods:**

306 patients with stage II and 205 patients with stage III colon cancer diagnosed between 2005 and 2016 who had undergone surgery in a curative setting were enrolled. Budding and TILs were assessed according to the criteria of the International Tumor Budding Consensus Conference (ITBCC) and the criteria of the International TILs Working Group (ITWG). Combinations of budding and TILs were analyzed, and the influence of adjuvant chemotherapy was assessed.

**Results:**

In stage II colon cancer, stratification into the four budding/TILs groups showed no significant differences in overall survival (OS) between the chemotherapy and the surgery-alone group, not even in cases with high-risk features. In stage III colon cancer, patients with low budding/high TILs benefited significantly from chemotherapy (*p*=0.005). Patients with high budding/low TILs as well as high budding/high TILs showed a trend to benefit from adjuvant treatment. However, no chemotherapy benefit was seen for the low budding/low TIL group.

**Conclusions:**

The budding/TIL combination identified subgroups in stage II and III colon cancer with and without benefit from adjuvant treatment. The results this study suggest that the combination of budding and TILs as tumor-host antagonists might be an additional helpful tool in adjuvant treatment decisions in stage II and III colon cancer.

## Introduction

Colorectal cancer (CRC) is one of the most common cancer types. In 2018, approximately 1.8 million people were newly diagnosed with CRC and about 880.000 people died from CRC worldwide [1]. Treatment regimes are based on the Tumor Node Metastasis (TNM) staging system, the grading according to the World Health Organization (WHO) classification, and molecular biomarkers [2]. However, in recent years, additive markers with the potential to predict prognosis or response to therapy or even being treatment targets have gained increasing attention.

Among them, on the tumor side, tumor budding, as a morphologic sign of the epithelial-mesenchymal transition (EMT), proved to be associated with T-stage, N-stage, vascular and lymphatic infiltration, local tumor recurrence, distant metastases, and higher tumor aggressiveness [3–14]. In 2016, criteria for evaluating and reporting of tumor budding in CRC on hematoxylin and eosin (H&E) stained slides have been well defined by the International Tumor Budding Consensus Conference (ITBCC) and have been validated afterward [11, 15]. Tumor budding is now accepted as an additional prognostic factor for CRC, according to the Union for International Cancer Control (UICC), and is listed as an essential and desirable diagnostic criterium for CRC in the current 5th edition of WHO Classification of Tumors [2, 16].

On the host immunity side, tumor-infiltrating lymphocytes (TILs) are also a popular object of interest in current research and have already reached therapeutic relevance in different human cancer types as an immunooncogenic target [17–20]. Increased TILs in CRC are an independent predictor of better prognosis [11, 21, 22]. The assessment of TILs on H&E stained slides has recently been standardized by the International TIL Working Group (ITWG), and its efficiency has currently been proven in a large series of 1034 CRC patients [23–25].

In our previous work, we could show that the combination of tumor budding and TILs is able to stratify patients with colon cancer into prognostic subgroups with different overall survival (OS). The parameter TILs proved to be more relevant regarding prognosis than the parameter budding. However, budding was also able to further stratify the low TIL cases into subgroups with different OS.

The aim of the present study was

1. to analyze the combination of budding and TILs, as assessed according to the ITBCC, respectively, ITWG criteria in stage II and III colon cancers with special focus on the influence of adjuvant chemotherapy
2. to identify “budding/TILs” subgroups that might impact adjuvant chemotherapeutic treatment decisions

## Material and methods

### Case selection

A search in our institutional database provided 306 cases of stage II colon cancer and 205 cases of stage III colon cancer, diagnosed between 2005 and 2016. All cases had undergone surgical treatment in a curative setting.

Cases with neoadjuvant treatment modalities and rectal carcinomas (due to high percentage of neoadjuvant treatment) were excluded from the study. Further patient and tumor characteristics are listed in Table 1.

**Table 1:** Summary of patient and tumor characteristics

Feature	Stage II (*n*=306)	Stage III (*n*=205)
	Frequency, *n* (%)	
Age (y; mean, *n*=511)	75 (47-97)	74 (36-97)
Sex (*n*=511)		
Male	154 (50.3)	98 (47.8)
Female	152 (49.7)	107 (52.2)
pT (*n*=511)		
pT1	0	6 (2.9)
pT2	2 (0.7)	16 (7.8)
pT3	254 (83.0)	120 (58.5)
pT4	50 (16.3)	63 (30.7)
pN (*n*=205)	---	
pN1	---	137 (66.8)
pN2	---	68 (33.2)
Tumor location (right/left, *n*=511)		
Right	214 (69.9)	132 (64.4)
Left	92 (30.1)	73 (35.6)
Grading (WHO 2019, *n*=511)		
Low grade	238 (77.8)	150 (73.2)
High grade	68 (22.2)	55 (26.8)
Venous invasion (*n*=511)		
V0	277 (90.5)	155 (75.6)
V1	28 (9.2)	50 (24.2)
V2	1 (0.3)	0
Lymphatic invasion (*n*=511)		
L0	241 (78.8)	76 (37.1)
L1	65 (21.2)	129 (62.9)
Mucinous (y/n; *n*=511)		
Mucinous	32 (10.5)	11 (5.4)
Not mucinous (NOS)	274 (89.5)	194 (94.6)
MMR status (*n*=50)		
MMR proficient	19 (57.6)	10 (58.8)
MMR deficient	14 (42.4)	7 (41.2)
RAS (*n*=65)		
Wild type	21 (77.8)	16 (42.1)
Mutated	6 (22.2)	22 (57.9)

Follow-up data were provided from the local tumor registry in Bayreuth. A complete follow-up was available for 477 cases. Median follow-up was 39 months (range 0-189 months). 247 patients were alive at study end, 110 died of disease, 66 died from other causes, and the cause of death was unknown in 34 patients.

The ethics commission of Friedrich-Alexander-University Erlangen-Nuremberg approved the study (study number 55_17 B).

### Histological evaluation

Hematoxylin and eosin-stained tumor slides of all patients were retrieved from our archives.

The slides were re-evaluated independently in terms of budding according to the criteria of the ITBCC by two different pathologists (CLS, BM) using an Olympus BX 53 (CLS), respectively, BX 46 (BM) microscope [14]. In brief, the most appropriate tumor slide was chosen (CLS), the invasion front was scanned for the hotspot area, and budding was counted in one hotspot (lens magnification 20×, ocular magnification 10×, eyepiece field number diameter 22). The number of buds was adjusted by the normalization factor as described. Budding was reported as proposed: low budding—0-4 buds (Bd1), intermediate budding—5-9 buds (Bd2), high budding >10 buds (Bd3). Only peritumoral budding at the invasive front was taken into account. Cases with intermediate (Bd2) and high budding (Bd3) were grouped together as one “high budding-group” as they had shown a trend to similar overall survival [11].

The percentage of tumor-associated lymphatic infiltration was semiquantitatively estimated on the same H&E stained slides by the two pathologists, according to the ITWG methodology [23, 24].

These included mononuclear inflammatory cells (lymphocytes and plasma cells) in the tumor-stromal compartment only, reported as a percentage of TILs as a continuous variable without focussing on hotspots. TILs outside the tumor borders and polymorphonuclear leukocytes were not taken into account. Areas of necrosis, fibrosis, and abscess formation were also excluded. The tumor slides were scanned in a 200 fold magnification (ocular ×10, objective ×20), and the average percentage amount of TILs was reported. Referring to our previous studies, a TILs cutoff at 5% served as the discrimination threshold between the “low TILs-group” (<5% TILs) and the “high TILs-group” (>5%) [11, 26].

**Fig. 1:**
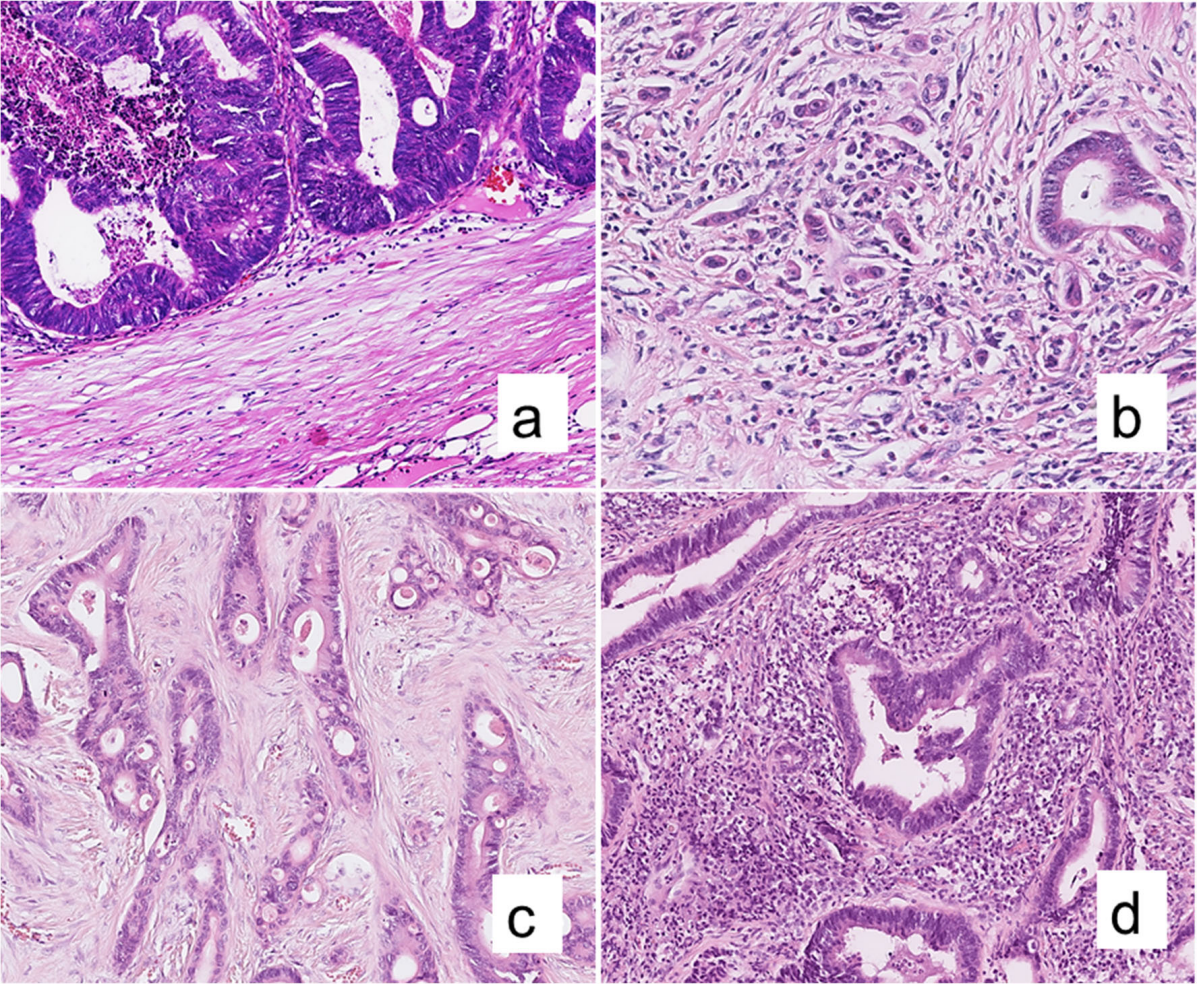
Representative histomorphological hematoxylin and eosin-stained example images for budding (a, b) and TILs (c, d) in colon cancer. Budding-images are taken from the hotspot at the tumor invasive front. TIL images show the central tumor area. (a) low budding (Bd1, 0 buds per hotspot, magnification 131×); (b) high budding (Bd3, 24 buds per hotspot, magnification 194×); (c) low TILs (magnification 147×); (d) high TILs (magnification 154×)

Representative histomorphological example images for low and high budding as well as low and high TILs are shown in Fig. 1.

Out of these results and according to our previous study, budding and TILs results were grouped as follows:

1. Low budding/high TILs (i.e., Bd1 + TILs >5%)
2. Low budding/low TILs (i.e., Bd1 + TILs<5%)
3. High budding/high TILs (i.e., Bd2 or Bd3 and TILs>5%)
4. High budding/low TILs (i.e., Bd2 or Bd3 and TILs<5%)

### Statistics

Statistical analyses were performed using the statistics program SPSS 21 (IBM Corp. Released 2012, IBM SPSS Statistics for Windows. Armonk, NY). Pearson’s chi-square test was used to test the relationship between different parameters. Interobserver agreement was tested by Cohen’s Kappa. Univariate survival analyses for overall survival (OS) were carried out using the Kaplan-Meier method with log-rank test. Multivariate survival analysis was performed using the Cox regression analysis. Hazard ratios and 95% confidence intervals (CI) were used to determine effect size. *p* values < 0.05 were considered statistically significant.

## Results

### Budding, TILs, and combination subgroups

Stage II colon cancer showed low budding in 234 (Bd1, 76.5%), intermediate budding in 56 (Bd2, 18.3%), and high budding in 16 cases (Bd3, 5.2%). One hundred fifty-two cases (49.7%) had <5% TILs and 154 cases (50.3%) had >5% TILs.

Stage III colon cancer showed low budding in 143 (Bd1; 69.8%), intermediate budding in 48 (Bd2; 28.4%), and high budding in 14 (Bd3; 6.8%) cases. One hundred and twenty-four cases (60.5%) had <5% TILs and 81 (39.5%) had >5% TILs.

Stage III tumors showed significantly more cases with <5%TILs than stage II tumors (*p*=0.010), whereas the distribution of Bd1–Bd3 was equal in both stages.

Higher budding was significantly associated with mucinous tumors (*p*=0.10), lymphatic vessel invasion (*p*=0.003), and venous invasion (*p*=0.021) and showed a trend to higher pT- (*p*=0.067) and pN-stages (*p*=0.076). Higher TILs were correlated with lower pN-stage (*p*=0.005), lower T-stage (*p*=0.011), non-mucinous tumors (*p*<0.001), and less lymphatic vessel invasion (*p*=0.003).

The combination of both parameters was correlated with pN (*p*=0.018), mucinous tumors (*p*=0.001), and lymphatic vessel invasion (*p*<0.001) and showed a trend with pT (*p*=0.054).

Interobserver agreement between the two pathologists was substantial for budding (*κ*=0.71, *p*<0.001) and fair for TILs (*κ*=0.246, *p*=0.001).

Table 2 shows the distribution of the cases among the four budding/TILs-groups.

**Table 2:** Distribution of the colon cancer cases of stage II and III among the four budding/TILs-groups

Budding/TIL group	Stage II (*n*, %)	Stage III (*n*, %)
Low budding/high TILs	116 (37.9)	55 (26.8)
Low budding/low TILs	107 (35.0)	77 (37.6)
High budding/high TILs	38 (12.4)	27 (13.2)
High budding/low TILs	45 (14.7)	46 (22.4)

**Fig. 2:**
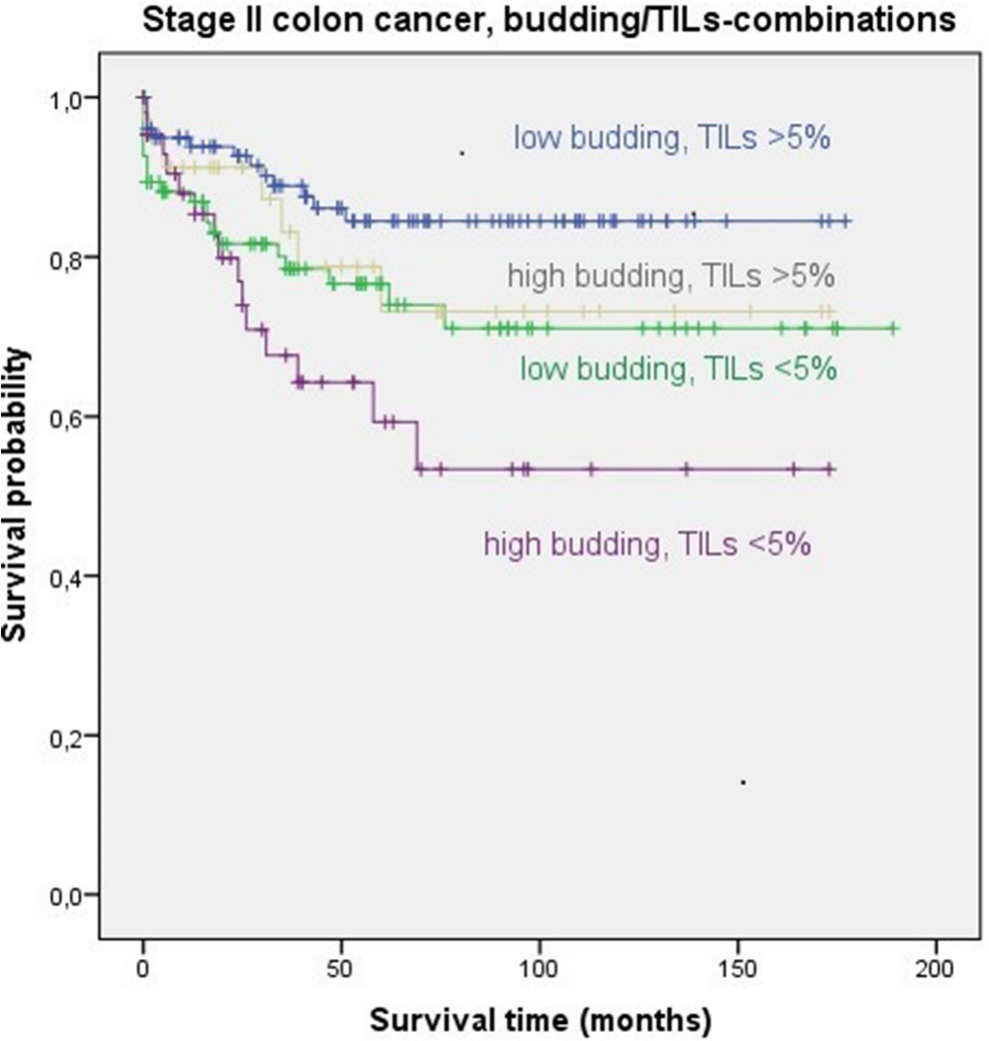
Kaplan-Meier survival analysis for all stage II colon cancer patients, stratified into the four budding/TIL groups. Differences between the low budding/high TILs group (blue) and the low budding/low TILs group (green) as well the high budding/high TILs group (purple) were statistically significant (*p*=0.047 and *p*=0.001, respectively)

In survival analysis, stage II tumors showed significant differences in OS survival between the low budding/high TILs-group versus the low budding/low TILs- (mean OS: 153.17 versus 141.25 months, 95%CI: 141.16-165.18 versus 123.26-159.23, respectively, *p*=0.047) and the high budding/low TILs-group (mean OS: 153.17 versus 105.77 months, 95%CI: 141.16-165.18 versus 136.06-155.94, respectively, *p*=0.001). Survival was best for the low budding/high TILs-group and worst for the high budding/low TILs-group. Cases with high budding/high TILs and low budding/low TILs showed almost equal OS-curves (mean OS: 134.45 versus 141.25 months, respectively, *p*=0.668). Kaplan-Meier survival curves for all stage II patients (with and without chemotherapy) are shown in Fig. 2.

**Fig. 3:**
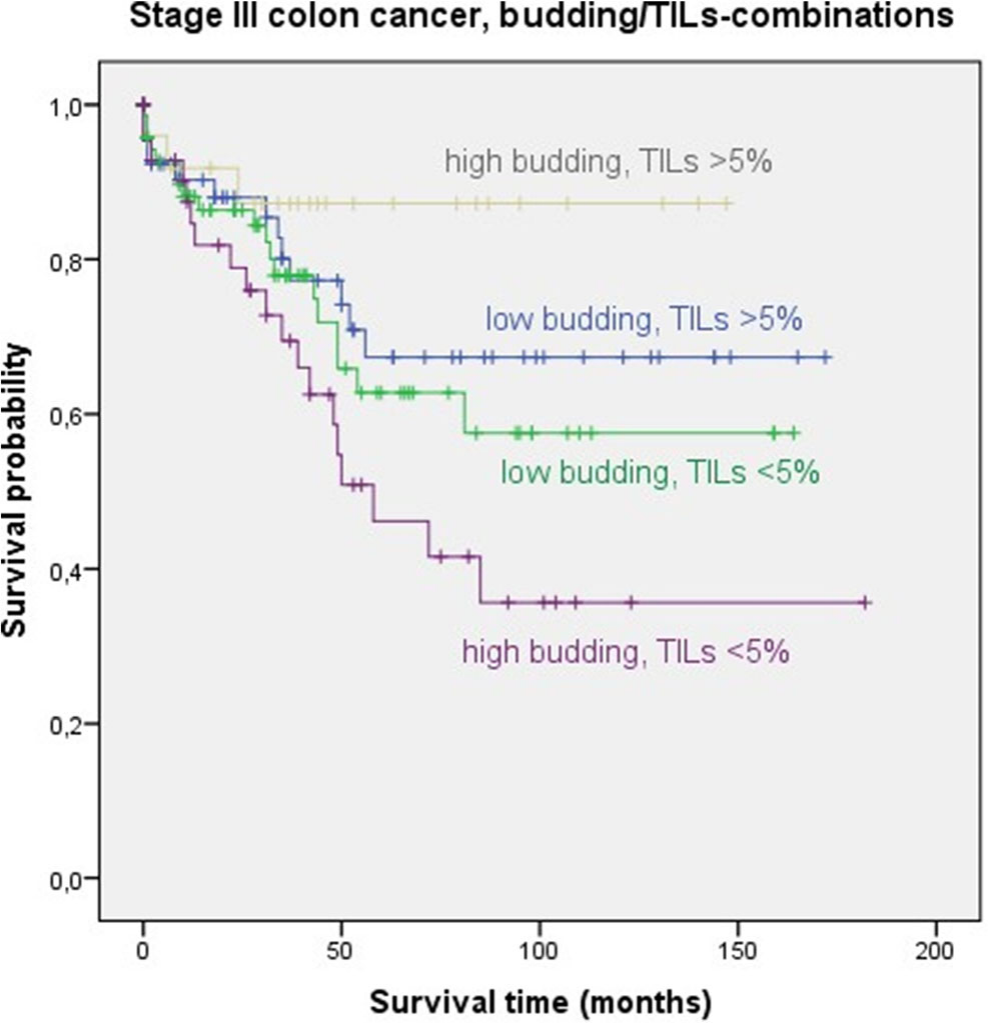
Kaplan-Meier survival analysis for all stage III colon cancer patients, stratified into the four budding/TIL groups. Differences between the low budding/high TILs group (blue) and the high budding/high TILs group (purple) as well as between the high budding/high TILs group (gray) and the high budding/low TILs group (purple) were statistically significant (*p*=0.036 and *p*=0.010, respectively)

Stage III tumors showed significant OS differences between the low budding/high TILs-group and the high budding/low TILs-group (mean OS: 125.40 versus 89.34 months, 95%CI: 104.09-146.72 versus 62.46-116.22, respectively, *p*=0.036) as well as between the high budding/high TILs-group and the high budding/low TILs-group (mean OS: 129.59 versus 89.34 months, 95%CI: 111.14-148.04 versus 62.46-116.22, respectively, *p*=0.010). Survival was best for the high budding/high TILs-group and worst for the high budding/low TILs-group. Kaplan-Meier survival curves for all stage III patients are shown in Fig. 3.

Interestingly, in stage III tumors, cases with high budding/high TILs showed superior OS survival compared to the low budding/high TILs group.

### Role of chemotherapy

Information about adjuvant chemotherapeutic treatment was available for 338 cases (66.2%; stage II: n=217, stage III: *n*=121). In stage II colon cancer, 28 (12.9%) patients received adjuvant chemotherapy and 189 (87.1%) did not. In stage III colon cancer, 68 (56.2%) patients received adjuvant chemotherapy and 53 (43.8%) did not. Information about the type of chemotherapy was available for 32 patients (33.3%). Among them, 19 patients were treated according to the FOLFOX scheme (folic acid, 5-fluorouracil, and oxaliplatin), 10 received 5-fluorouracil as monotherapy, and 3 received FOLFOX in combination with capecitabine.

Patients with chemotherapy in stage II were significantly more often high grade (*p*=0.003), higher pT-stages (*p*=0.001), and had more often positive surgical margins (*p*=0.002), whereas no significant correlation was found with lymphatic vessel invasion, venous invasion, budding, and TILs. Three out of 14 patients in stage II received chemotherapy despite microsatellite instability against one out of 18 patients in the microsatellite stable group.

Chemotherapy in stage III cases was significantly correlated with pN-stage (*p*=0.047) and lymphatic vessel invasion (*p*=0.027). No significant correlation was found with the other parameters. Reasons for cases that did not receive adjuvant treatment in stage III were mainly multiple or limiting comorbidities, patients’ age or patients’ decision.

In Kaplan-Meier analysis, no difference in OS survival was found between patients with and without chemotherapy in stage II (*p*=0.653; mean survival without chemotherapy: 148.09 months, 95% CI: 136.03 - 160.16 months; mean survival with chemotherapy: 145.92 months, 95% CI: 121.55–170.29 months), whereas patients with stage III colon cancer benefited significantly from adjuvant treatment (*p*=0.006, mean survival without chemotherapy: 90.17 months, 95% CI: 65.65–114.69 months; mean survival with chemotherapy: 128.94 months, 95% CI: 111.34–146.54 months).

Concerning the budding/TILs-combination in stage II, no obvious difference in OS was found between cases with and without chemotherapy in the low budding/high TILs-group as well as the low budding/low TILs-group, whereas the other two groups showed differences between the chemo- versus non-chemo-group. However, these differences were not statistically significant.

In stage II colon cancer, Pearson’s Chi-square test between the four budding/TILs combinations and the clinicopathological parameters showed significant higher pT-stages and more high-grade cases in the chemotherapy group for the low budding/high TIL group (*p*=0.005 for pT and 0.035 for grading) as well as the low budding/low TILs group (*p*<0.001 for pT and <0.001 for grading). No correlations were found with the other parameters as well as for the other two budding/TIL groups. Correlations between the four budding/TILs groups in stage II colon cancer and clinicopathological parameters with and without chemotherapy are shown in Table 3.

**Table 3:** Correlation analysis between the four budding/TIL groups and clinicopathological parameters in stage II colon cancer with and without chemotherapy

Feature (*n*/%)	Low buds/high TILs (*n*=103)			Low buds/low TILs (*n*=96)			High buds/high TILs (*n*=35)			High buds/low TILs (*n*=44)		
	CTH yes/no (*n*=77)		*p* value	CTH yes/no (*n*=81)		*p* value	CTH yes/no (*n*=25)		*p* value	CTH yes/no (*n*=34)		*p* value
	Yes (14/18.2)	No (63/81.8)		Yes (7/8.64)	No (74/91.4)		Yes (3/12)	No (22/88)		Yes (4/11.8)	No (30/88.2)	
pT stage			*0.005*			*<0.001*			0.604			0.481
1	0	0		0	0							
2	0	1 (100.0)		0	0					0	1 (100.0)	
3	9 (13.6)	57 (86.4)		2 (3.0)	64 (97.0)		3 (13.0)	20 (87.0)		4 (14.8)	23 (85.2)	
4	5 (50.0)	5 (50.0)		5 (33.3)	10 (66.7)		0	2 (100.0)		0	6 (100.0)	
Mucinous			0.243			0.603			0.701			0.522
Yes	1 (50.0)	1 (50.0)		1 (5.6)	17 (94.4)		0	0		0	3 (100.0)	
No	13 (17.3)	62 (82.7)		6 (9.5)	57 (90.5)		3 (12.0)	22 (88.0)		4 (12.9)	27 (87.1)	
Grading (WHO)			*0.035*			*<0.001*			0.604			0.292
Low (G1-2)	8 (13.1)	53 (86.9)		2 (3.1)	62 (96.9)		3 (13.0)	20 (87.0)		4 (14.8)	23 (85.2)	
High (G3)	6 (37.5)	10 (62.5)		5 (29.4)	12 (70.6)		0	2 (100.0)		0	7 (100.0)	
Venous invasion (V)			0.215			0.440			0.720			0.292
0	11 (16.2)	57 (83.8)		7 (9.3)	68 (90.7)		3 (12.5)	21 (87.5)		4 (14.8)	23 (85.2)	
1	3 (33.3)	6 (66.7)		0	6 (100.0)		0	1 (100.0)		0	7 (100.0)	
Lymphatic vessel invasion (L)			0.094			0.968			0.701			0.576
0	10 (15.2)	56 (84.8)		6 (8.7)	63 (91.3)		2 (10.5)	17 (89.5)		3 (14.3)	18 (85.7)	
1	4 (36.4)	7 (63.6)		1 (8.3)	11 (91.7)		1 (16.7)	5 (83.3)		1 (7.7)	12 (92.3)	

*p*-values in italics are statistically significant

**Fig. 4:**
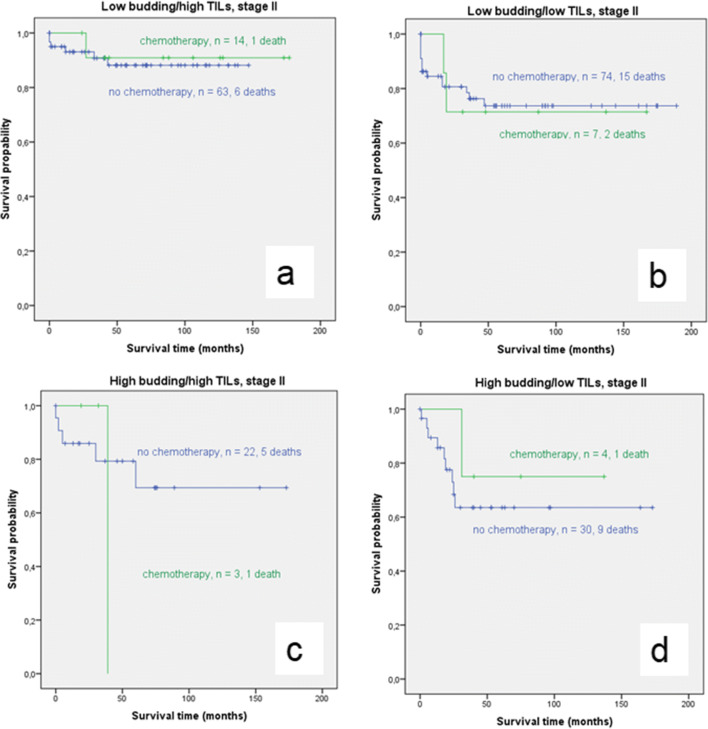
Kaplan-Meier survival analysis in stage II colon cancer for each of the four budding/TIL groups (blue: no chemotherapy, green: chemotherapy). Differences between patients with and without chemotherapy were not statistically significant

Kaplan-Meier survival analysis in stage II colon cancer for each of the four budding/TILs groups separately is shown in Fig. 4. No significant difference in OS between cases with and without chemotherapy was found in each group. Most notably, no difference in OS between cases with and without adjuvant treatment was found in the low budding/high TIL group and the low budding/low TIL group, although these groups showed significant differences between high-risk cases and non-high-risk cases for pT and grading with and without chemotherapy.

In stage III colon cancer, Pearson’s Chi-square test between the four budding/TIL groups and the clinicopathological parameters with and without chemotherapy showed significant higher pN-stages for the high budding/low TIL group (*p*=0.029) and significant more high-grade cases in the low budding/high TIL group (*p*=0.005). Cases with low budding/low TILs showed significantly more often lymphatic vessel invasion (*p*=0.040). No correlation was found with the other parameters. The results of the correlation analyses between the four budding/TILs groups in stage III colon cancer and clinicopathological parameters with and without chemotherapy are shown in Table 4.

**Table 4:** Correlation analysis between the four budding/TIL groups and clinicopathological parameters in stage III colon cancer with and without chemotherapy

Feature (*n*/%)	Low buds/high TILs (*n*=55)			Low buds/low TILs (*n*=77)			High buds/high TILs (*n*=27)			High buds/low TILs (*n*=46)		
	CTH yes/no (*n*=33)		*p* value	CTH yes/no (*n*=48)		*p* value	CTH yes/no (*n*=15)		*p* value	CTH yes/no (*n*=25)		*p* value
	Yes (22/66.7)	No (11/33.3)		Yes (24/50)	No (24/50)		Yes (7/46.7)	No (8/53.3)		Yes (15/60.0)	No (10/40.0)	
pT stage			0.755			0.572			0.743			0.135
1	2 (100.0)	0		0	0		0	1 (100.0)				
2	2 (66.7)	1 (33.3)		1 (50.0)	1 (50.0)		2 (100.0)	0		1 (50.0)	1 (50.0)	
3	12 (60.0)	8 (40.0)		18 (52.9)	16 (47.1)		4 (44.4)	5 (55.6)		4 (40.0)	6 (60.0)	
4	6 (75.0)	2 (25.0)		5 (41.7)	7 (58.3)		1 (33.3)	2 (66.7)		10 (76.9)	3 (23.1)	
pN stage			0.299			0.762			0.926			*0.029*
1	14 (60.9)	9 (39.1)		16 (48.5)	17 (51.5)		6 (46.2)	7 (53.8)		5 (38.5)	8 (61.5)	
2	8 (80.0)	2 (20.0)		8 (53.3)	7 (46.7)		1 (50.0)	1 (50.0)		8 (83.3)	2 (16.7)	
Mucinous			0.211			0.323			---			0.400
Yes	1 (33.3)	2 (66.7)		0	1 (100.0)		0	0		0	1 (100.0)	
No	21 (70.0)	9 (30.0)		24 (51.1)	23 (48.9)		7 (46.7)	8 (53.3)		15 (62.5)	9 (37.5)	
Grading (WHO)			*0.005*			0.097			0.733			0.607
Low (G1-2)	9 (47.4)	10 (52.6)		20 (57.1)	15 (42.9)		6 (46.2)	7 (53.8)		10 (58.8)	7 (41.2)	
High (G3)	13 (92.9)	1 (7.1)		4 (30.8)	9 (69.2)		1 (50.0)	1 (50.0)		5 (62.5)	3 (37.5)	
Venous invasion (V)			0.508			0.500			0.200			0.654
0	18 (64.3)	10 (35.7)		16 (48.5)	17 (51.5)		5 (38.5)	8 (61.5)		13 (59.1)	9 (40.9)	
1	4 (80.0)	1 (20.0)		8 (53.3)	7 (46.7)		2 (100.0)	0		2 (66.7)	1 (33.3)	
Lymphatic vessel invasion (L)			0.086			*0.040*			0.573			0.545
0	7 (50.0)	7 (50.0)		7 (33.3)	14 (66.7)		2 (40.0)	3 (60.0)		4 (66.7)	2 (33.3)	
1	15 (78.9)	4 (21.1)		17 (63.0)	10 (37.0)		5 (50.0)	5 (50.0)		11 (57.9)	8 (42.1)	

*p*-values in italics are statistically significant

**Fig. 5:**
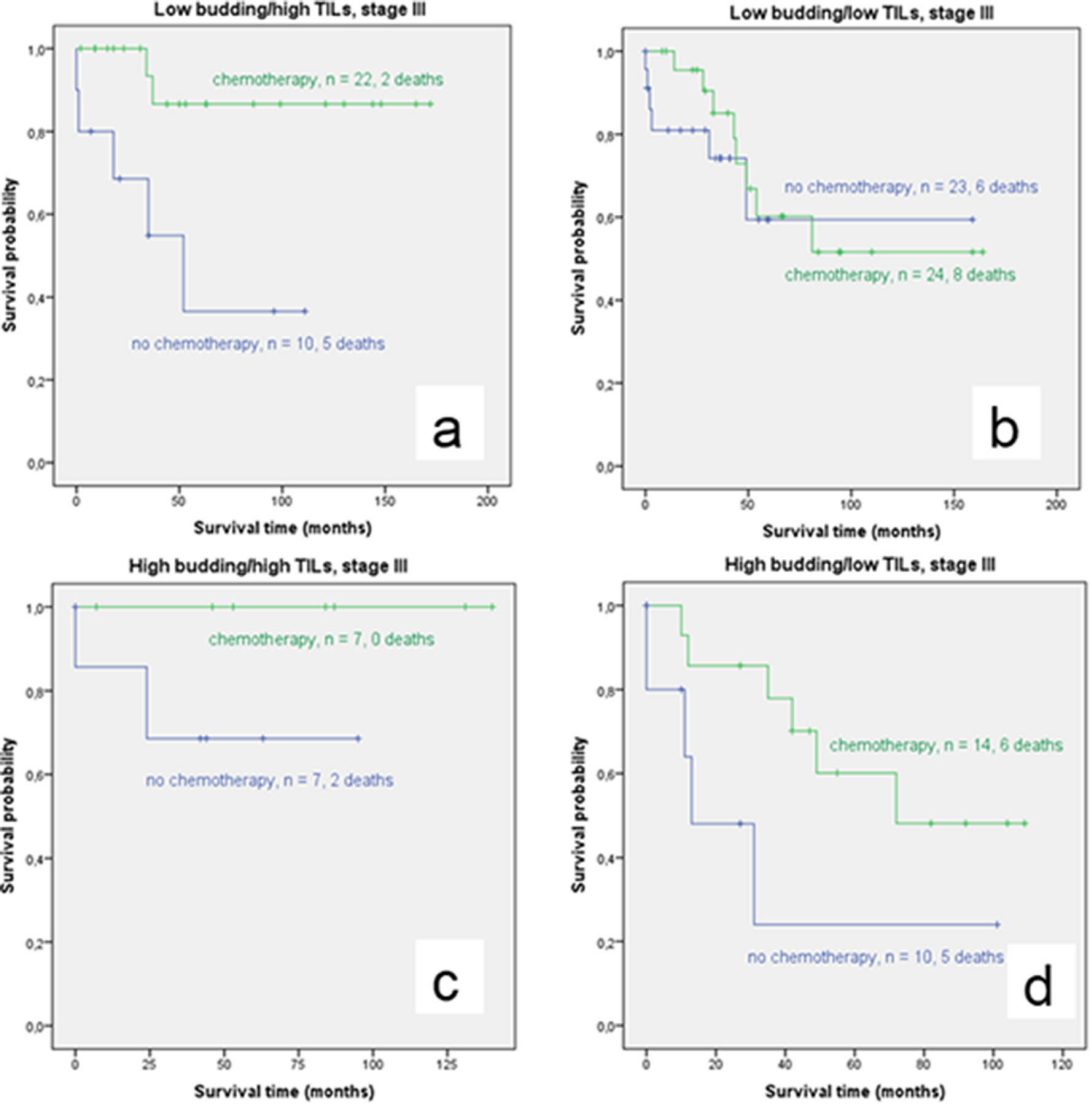
Kaplan-Meier survival analysis in stage III colon cancer for each of the four budding/TIL groups (blue: no chemotherapy, green: chemotherapy). The difference in OS between the chemotherapy versus non-chemotherapy group is significant in the low budding/high TILs group ((a) *p*=0.005) and showed a trend to a chemotherapy benefit in the high budding/low TIL group ((d) *p*=0.063). The difference between both groups was not significant in the high budding/high TIL group ((c) *p*=0.138). No difference in OS with and without chemotherapy was seen in the low budding/low TIL group ((b) *p*=0.550)

Kaplan-Meier survival analysis in stage III colon cancer for each of the four budding/TILs groups separately is shown in Fig. 5. Patients with low budding/high TILs benefited significantly from adjuvant treatment (*p*=0.005). This group showed significantly more high-grade cases in the chemotherapy group than in the group without chemotherapy. No significant difference in patients with and without chemotherapy was seen in the low budding/low TILs group (*p*=0.550). Patients with high budding/high TILs showed better OS with chemotherapy. However, the difference was not significant (*p*=0.138). In patients with high budding/low TILs, a trend to benefit from chemotherapy was seen (*p*=0.063). This group had significantly more cases with a higher pN-stage in the chemotherapy cohort.

Table 5 shows the survival time in months with 95%CI for the budding/TILs-combinations with and without chemotherapy in stage II and stage III colon cancer.

**Table 5:** Survival time in months with 95%CI for the budding/TILs-combinations with and without chemotherapy in stage II and stage III colon cancer

Tumor-stage (UICC)	Score budding/TILs	Chemotherapy yes/no (*n*, %)				*p* value
		Yes		No		
		*n* (%)	Survival time (months, mean, 95%CI)	*n* (%)	Survival time (months, mean, 95%CI)	
II	Low buds/high TILs (*n*=73)	12 (16.4)	131.69 (120.11-143.27)	61 (83.6)	163.36 (137.88-188.85)	0.770
	Low buds/low TILs (*n*=74)	7 (9.5)	124.43 (74.56-174.30)	67 (90.5)	142.73 (122.26-163.21)	0.928
	High buds/high TILs (*n*=25)	3 (12.0)	39.0 (39.0-39.0)	22 (88.0)	128.31 (94.41-162.21)	0.586
	High buds/low TILs (*n*=34)	4 (11.8)	110.50 (65.52-155.48)	30 (88.2)	115.80 (85.51-146.09)	0.546
III	Low buds/high TILs (*n*=32)	22 (68.8)	153.80 (130.32-177.28)	10 (31.3)	57.06 (26.17-87.95)	*0.005*
	Low buds/low TILs (*n*=47)	24 (51.1)	107.25 (77.99-136.50)	23 (48.9)	75.58 (51.68-99.48)	0.550
	High buds/high TILs (*n*=14)	7 (50.0)	122.60 (92.10-153.10)	7 (50.0)	53.50 (11.99-95.01)	0.138
	High buds/low TILs (*n*=24)	14 (58.3)	73.55 (52.35-94.76)	10 (41.7)	35.52 (3.78-67.26)	0.063

*p*-values in italics are statistically significant

Comparison of relative risk with and without chemotherapy for each of the budding/TILs-groups is shown in Table 6.

**Table 6:** Comparison of relative risk for each of the budding/TIL groups in stage II and III colon cancer with and without chemotherapy

Tumor stage (UICC)	Score budding/TILs	Chemotherapy	Hazard ratio (95% CI)	*p* value
II	Low buds/high TILs (*n*=73)	Yes	1.0	0.772
		No	0.731 (0.088-6.079)	
	Low buds/low TILs (*n*=74)	Yes	1.0	0.929
		No	1.070 (0.244-4.688)	
	High buds/high TILs (*n*=25)	Yes	1.0	0.591
		No	1.826 (0.203-16.441)	
	High buds/low TILs (*n*=34)	Yes	1.0	0.552
		No	0.533 (0.067-4.242)	
III	Low buds/high TILs (*n*=32)	Yes	1.0	*0.017*
		No	0.134 (0.026-0.696)	
	Low buds/low TILs (*n*=47)	Yes	1.0	0.554
		No	0.721 (0.244-2.130)	
	High buds/high TILs (*n*=14)	Yes	1.0	0.462
		No	0.014 (0-1234,265)	
	High buds/low TILs (*n*=24)	Yes	1.0	0.076
		No	0.322 (0.092-1.125)	

*p*-values in italics are statistically significant

**Fig. 6:**
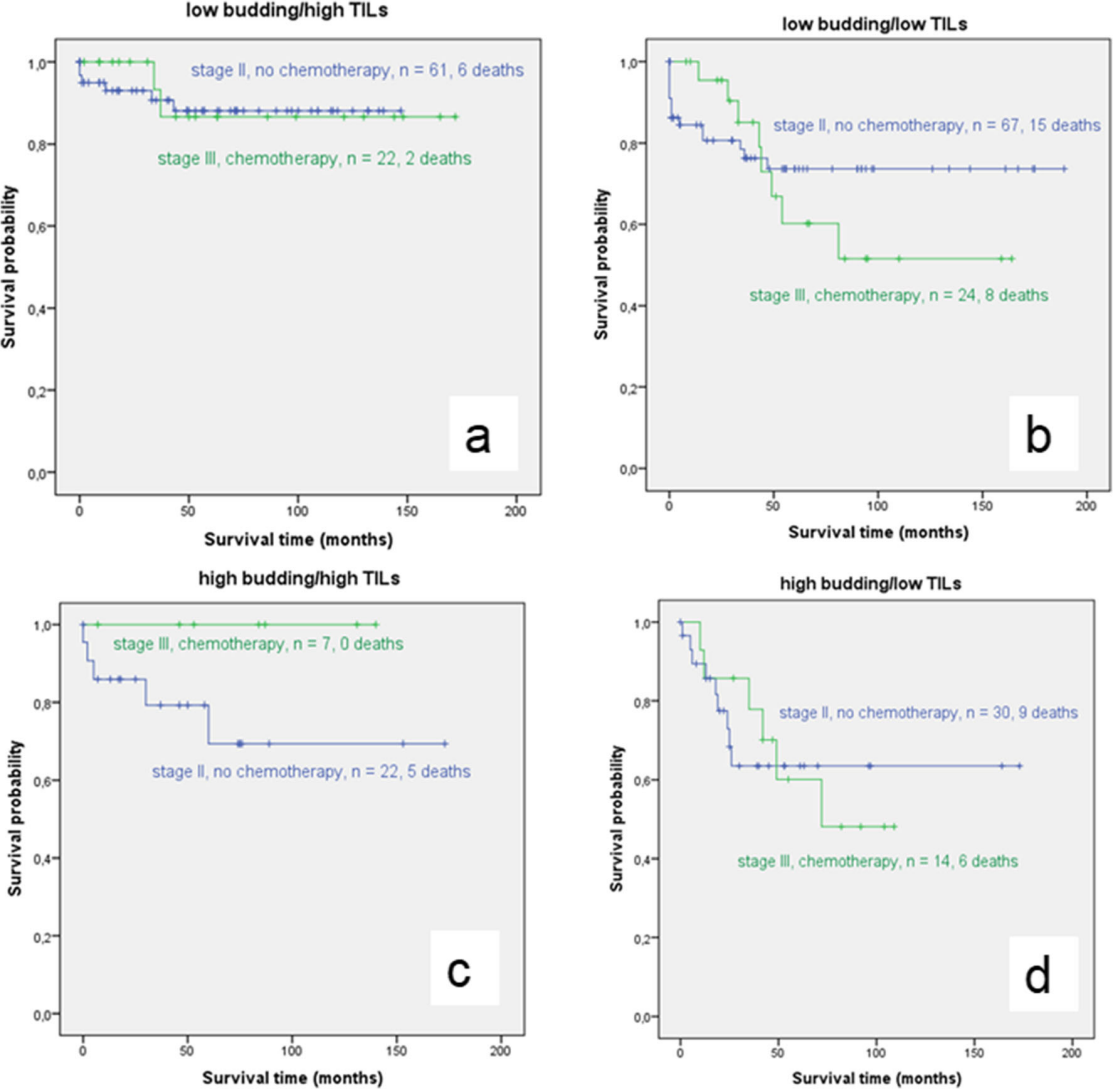
Kaplan-Meier survival analysis of stage II-cases without chemotherapy versus stage III-cases with chemotherapy for each of the four budding/TIL groups (blue: no chemotherapy, green: chemotherapy). No statistical significant differences were found between both groups. Most notably, OS was almost similar for stage II-cases without chemotherapy and stage III-cases with chemotherapy in the low budding/high TILs group, emphazising the high chemotherapeutic benefit for this budding/TIL subgroup in stage III ((a) *p*=0.878)

When all stage II cases without adjuvant treatment were compared to all stage III cases with adjuvant treatment, OS was better for the stage II-cases. However, this difference was not statistically significant (*p*=0.924). In separate analyses for each budding/TIL group, stage III cases with chemotherapy showed almost equal survival to stage II cases without chemotherapy in the low budding/high TIL group (mean OS for stage II: 131,69 months, 95%CI: 120.11-143.27 versus stage III:153.80, 95%CI: 130.32-177.84; *p*=0.878). The low budding/low TIL group and the high budding/low TIL group showed better survival for stage II colon cancer compared to stage III but no significant differences were found (*p*=0.581 and *p*=0.964). The high budding/high TIL group was the only group in which the stage III cases with chemotherapy showed superior OS compared to the stage II cases without chemotherapy. However, this difference was also not significant (*p*=0.158, Fig. 6).

## Discussion

In recent years, tumor budding and TILs have come into the focus of interest in CRC research. On the tumor side, tumor budding as a morphological sign of the epithelial-mesenchymal transition at the tumor invasive margin has been shown to be correlated with T-stage, N-stage, M-stage, vascular and lymphatic invasion, local tumor recurrence, and higher tumor aggressiveness [3–13, 27, 28]. On the host-immunity side, TILs as part of the tumor microenvironment have already found their way into current treatment regimes in different tumor types, including melanoma, lung cancer, or breast cancer [17–20]. A higher amount of TILs has been associated with longer patient survival in a number of different malignancies [11, 24, 29, 30].

An approach of a combination of a marker of the tumor microenvironment and a marker of host immunity has recently been published by Cha et al. [31]. They used a combination of TILs (as assessed by the Klintrup-Mäkinen method) and the neutrophil-to-lymphocyte ratio to stratify stage III colorectal cancer for adjuvant treatment decision which allowed a more detailed prognostic stratification compared to the stratification by TILs alone. Additionally, van Wyk et al. found a significant association between tumor budding and the tumor microenvironment as assessed by the Klintrup-Mäkinen grade and tumor stroma percentage combined as the Glasgow Microenvironment Score (GMS) in their study on H&E stained slides without additional immunohistochemistry [32]. The role of the budding-TILs interaction as “pro-/anti-tumor” model was first proposed by Lugli et al. in 2009 [33]. In their series of 300 cases with double immunostaining for CD8 (as marker for TILs) and CK22 (as marker for budding), patients with a high CD8+/buds index demonstrated more favorable features compared to the low CD8+/buds index patients.

We could recently confirm these results in two of our own H&E studies on large series of 501 and 576 CRC patients [11, 14]. The budding-TIL combination as tumor-host antagonists was not only able to stratify stages I-IV CRC of all grades but especially the large amount of WHO low-grade CRC into different subgroups with impact on OS. Most notably, the “factor TILs” proved to be a stronger predictor of better OS survival than the “factor budding.” Nevertheless, budding was able to further stratify the low TIL subgroups into two subgroups with significantly different OS.

Until recently, assessment of budding and TILs has been done in many different ways with or without the help of additional methodologies (like immunohistochemistry or computer-assisted assessment), making a comparison of study results difficult. However, in the meantime, the criteria for assessment of both features have been standardized and validated on H&E stained slides with international consensus each [15, 23–25, 34]. Interobserver agreement in our study was substantial for the parameter “budding” but only fair for the parameter “TILs” between the two pathologists. Fuchs et al. found a moderate interobserver agreement for TIL assessment between two pathologists without intensive training (*n* = 181, *κ* = 0.436), which could be improved to good interobserver agreement (*n* = 100, *κ* = 0.753) after face-to-face training of the ITWG-method [25].

In stage II CRC, the role of adjuvant chemotherapy is not definitely clear yet. Most patients do not benefit from adjuvant treatment but it can be offered optionally in “high risk” cases, i.e., pT4, poor differentiation, lymphovascular or perineural invasion, bowel obstruction, tumor perforation, and positive margins or insufficient number of lymph nodes [35, 36]. As tumor budding has been shown to be an indicator of shorter disease-free survival in stage II colorectal cancer, it was proposed to include tumor budding among the high-risk factors reported [4, 15]. Therefore, tumor budding has been adopted as a potential tumor-related prognostic factor by the UICC [2, 16]. Recently, the results from the SACURA Trial, a prospective multicenter study on the prognostic and predictive impact of tumor budding on a large cohort of 991 cases of stage II colon cancer, revealed a tendency beneficial effect of adjuvant chemotherapy for tumors with intermediate and high budding (Bd2 respectively Bd3 according to ITBCC) but the effect was not significant [37]. This is in analogy to our results, where no significant difference in OS between cases with and without chemotherapy was found in each budding/TIL group for stage II colon cancer. Most notably, no differences in OS between cases with and without adjuvant treatment were found in the low budding/high TIL group and the low budding/low TILs group, although these groups showed significantly more high-risk cases (higher pT and high grade) in their chemotherapy arm, leading to the conclusion, that those cases might not benefit from adjuvant treatment, even in case of high-risk features. As cases with low budding/low TILs show worse OS survival compared to the low budding/high TILs group in stage II tumors, the use of adjuvant treatment for high-risk cases in this group remains the subject of future studies.

Lee et al. found a correlation of tumor budding (assessed according to the ITBCC criteria) with pT4 and lymphovascular invasion as well as 5-year disease-specific survival (DSS) and OS in their 135 stage II colon cancer cohort. Survival curves could be further stratified by the combination of budding and poorly differentiated cluster (PDC) [38]. In accordance with these results, our study revealed a trend for higher budding to higher pT- and pN-stages (*p*=0.067 and *p*=0.076, respectively). Higher budding was also significantly associated with lymphatic vessel invasion (*p*=0.003) and venous invasion (*p*=0.021). According to the results of a systematic review and pooled analysis of 12 studies that included a total of 1652 patients by Petrelli et al., tumor budding was associated with worse survival in stage II CRC, in particular in pT3 tumors, who might benefit from adjuvant treatment [36].

Patients with stage III colorectal cancer are treated with adjuvant chemotherapy according to current guidelines. The FOLFOX regimen (folinic acid, 5-fluorouracil, and oxaliplatin) or alternatively the XELOX regimen (oxaliplatin and capecitabine) is regarded as the current standard of care [35]. Interestingly, even if adjuvant treatment in stage III is the current standard, Yamadera et al. reported for two independent cohorts of 203, respectively, 346 cases of CRC a better cancer-specific survival for patients with adjuvant treatment compared to the surgery-alone groups in low budding tumors, whereas patients with high-budding tumors did not show a benefit from chemotherapy [39].

In our study, however, stage III cases with high budding/high TILs showed superior OS survival compared to the low budding/high TIL group, which showed the best OS in our previous studies (stages I to IV pooled) [11, 14]. In fact, the stage III high budding/high TILs cases were the only group that was even superior in OS when compared to the stage II cases without chemotherapy, even if this difference was not significant. This might lead to the conclusion that buds might be a good chemotherapeutic target but is in contrast to the results of the study by Yamadera et al. [39].

Patients with low budding/high TILs benefited significantly from adjuvant treatment (*p*=0.005) and reached almost similar OS survival rates as patients with stage II colon cancer without adjuvant treatment (no difference in OS, *p*=0.878). This group showed significantly more high-grade cases in the chemotherapy group than in the group without chemotherapy. No significant difference in patients with and without chemotherapy was seen in the low budding/low TIL group (*p*=0.550). This leaves room for the conclusion that patients with low budding/low TILs might not benefit from adjuvant chemotherapy.

A limitation of our study is the fact that molecular data (e.g., MMR status, RAS, and RAF) were only available for a small number of stage II and stage III cancers. This can mainly be explained by the fact that RAS- and RAF-mutation status is most important for treatment decisions in stage IV colorectal cancer, and MMR-status analysis was until recently only mandatory in case of positive Amsterdam—or revised Bethesda-criteria [40]. Higher amounts of TILs are known to be associated with cancers showing microsatellite instability. However, TILs have also been shown to have important prognostic value in all colorectal carcinomas, regardless of MSI status [41]. As for budding, the role of its association with MSI is not definitely clear yet. Eriksen et al. found a near significant association between low-grade tumor budding and MSI status in 573 cases of stage II colon cancer (*p*=0.079) [42]. However, recently published series of 342 and 215 colorectal cancer cases by Dawson et al. and van Wyk et al. could not show an association between MSI-status and tumor budding (*p*=0.388 and *p*=0.592, respectively) [4, 34].

## Conclusion

Assessment of budding and TILs on H&E stained slides is a reproducible, cost-effective, and time-saving method with a proven impact on patient outcome and treatment strategies. Both features can be simply assessed in routine practice without the need of additional methods, even in the modern molecular era. Our study shows that the combination of budding and TILs has the potential to identify patient subgroups in stage II and III colon cancer without adjuvant treatment benefit. This raises the question if potential overtreatment with adversal side effects of a chemotherapy could possibly be avoided in these subgroups. On the other hand, it underlines the fact that patients with low budding/high TILs and high budding/low TILs in stage III colon cancer benefit significantly from adjuvant treatment. Even if the number of cases is limited in our study, we could show that the budding/TIL combination as tumor-host antagonists in stage II and III colon cancer patients has the potential to predict outcome in the issue of adjuvant treatment. To the best of our knowledge, this study is the first that analyses the influence of both, budding and TILs and their combination as tumor-host antagonists on colon cancer, stages II and III with a focus on the influence of adjuvant chemotherapy.

## Data Availability

Data, as far as not anyway shown, are available on demand from the corresponding author via e-mail: Corinna.Lang-Schwarz@klinikum-bayreuth.de.
